# The pharmaco-epigenetics of hypertension: a focus on microRNA

**DOI:** 10.1007/s11010-024-04947-9

**Published:** 2024-02-29

**Authors:** Serge Yaacoub, Ammar Boudaka, Ali AlKhatib, Gianfranco Pintus, Amirhossein Sahebkar, Firas Kobeissy, Ali H. Eid

**Affiliations:** 1 Faculty of Medicine, American University of Beirut, Beirut, Lebanon; 2 Department of Basic Medical Sciences, College of Medicine, QU Health, Qatar University, Doha, Qatar; 3 Department of Nutrition and Food Sciences, Lebanese International University, Beirut, Lebanon; 4 Department of Biomedical Sciences, University of Sassari, Viale San Pietro, 07100 Sassari, Italy; 5 Biotechnology Research Center, Pharmaceutical Technology Institute, Mashhad University of Medical Sciences, Mashhad, Iran; 6 Applied Biomedical Research Center, Mashhad University of Medical Sciences, Mashhad, Iran; 7 Department of Neurobiology, Center for Neurotrauma, Multiomics and Biomarkers (CNMB), Morehouse School of Medicine, Neuroscience Institute, Atlanta, GA USA

**Keywords:** Cardiovascular disease, Endothelium, Blood pressure, Antihypertensive response, Aspirin, Clopidogrel, Statins

## Abstract

Hypertension is a major harbinger of cardiovascular morbidity and mortality. It predisposes to higher rates of myocardial infarction, chronic kidney failure, stroke, and heart failure than most other risk factors. By 2025, the prevalence of hypertension is projected to reach 1.5 billion people. The pathophysiology of this disease is multifaceted, as it involves nitric oxide and endothelin dysregulation, reactive oxygen species, vascular smooth muscle proliferation, and vessel wall calcification, among others. With the advent of new biomolecular techniques, various studies have elucidated a gaping hole in the etiology and mechanisms of hypertension. Indeed, epigenetics, DNA methylation, histone modification, and microRNA-mediated translational silencing appear to play crucial roles in altering the molecular phenotype into a hypertensive profile. Here, we critically review the experimentally determined associations between microRNA (miRNA) molecules and hypertension pharmacotherapy. Particular attention is given to the epigenetic mechanisms underlying the physiological responses to antihypertensive drugs like candesartan, and other relevant drugs like clopidogrel, aspirin, and statins among others. Furthermore, how miRNA affects the pharmaco-epigenetics of hypertension is especially highlighted.

## Introduction

The evolution of hypertension is multifactorial [1–4]. Most hypertension cases worldwide are due to primary causes, i.e. essential hypertension [5]. A smaller subset of hypertensive individuals around the world suffers from secondarily induced hypertension [6]. The most common causes of secondary hypertension include coarctated aorta, renal parenchymal diseases, renovascular diseases, endocrine disorders, pregnancy-induced hypertension, drug-related hypertension, and sleep apnea [6].

The pathophysiological basis of hypertension is multifactorial [7]. Dysregulation of pressure natriuresis via excessive sympathetic nervous system stimulation, impaired kidney functionality, and improper hormonal activation of salt and water excretion regulators can alter vascular tone and thus predispose to a hypertensive state [8, 9]. Moreover, the vascular endothelium provides homeostasis in the cardiovascular system [10]. This is attained by the incessant release of elements that act to modulate smooth muscle cell contraction, cellular proliferation, the aggregation of platelets, and vascular wall permeability [11]. The dysfunction of this endothelium is due to the imbalance of its regulatory elements [10]. This has a pertinent effect on the development of various diseases, including hypertension. This endothelial dysfunction, in its chronic form, leads to considerable vascular wall remodeling. Moreover, it impacts blood pressure regulation in the context of hypertension [10]. The functionality of this endothelial system has been widely targeted by drug therapies in the context of various disease states [12]. With regards to hypertension, this includes inhibitors of the renin-angiotensin aldosterone system (RAAS), in addition to other therapies like statins and antioxidants [10]. Other complementary approaches like herbal medicine have also been employed in the war against cardiovascular disease in general or hypertension in particular [13–17]. Importantly, these diverse approaches all support the vital role of the endothelial system in hypertension development.

**Fig. 1:**
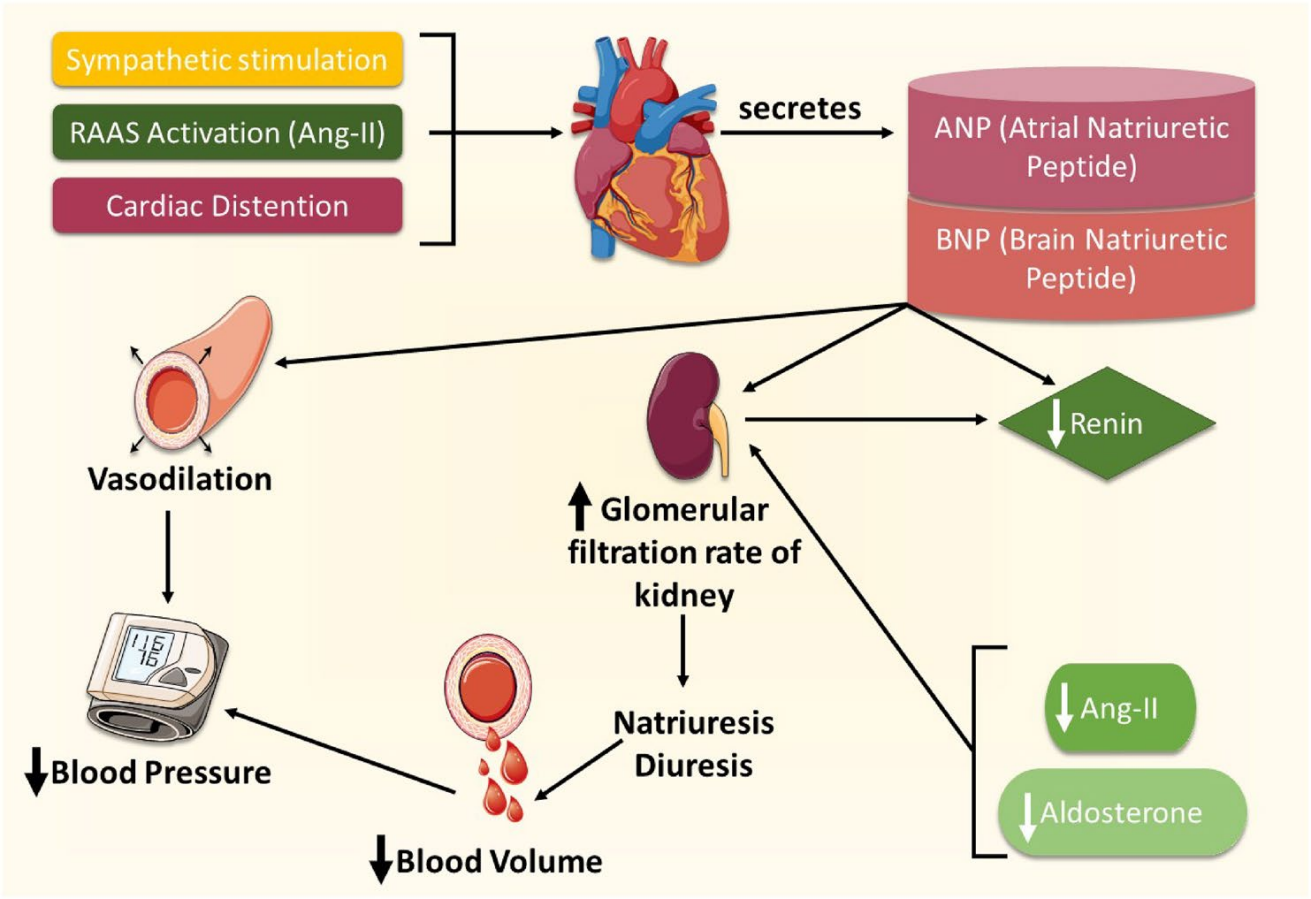
The interplay between the sympathetic nervous system (SNS) and renin-angiotensin aldosterone system (RAAS) in altering cardiovascular hemodynamics. SNS and RAAS activation induce the heart to secrete protective mediators (atrial natriuretic and brain natriuretic peptides) capable of ameliorating a hypertensive state

The onset and pathogenesis of hypertension involve several molecular and cellular parameters. Hence, a better understanding of the molecular basis of hypertension is vital for generating new treatment modalities. Many players like reactive oxygen species (ROS), endothelin, and vascular endothelial growth factor (VEGF) are among the key molecules implicated in this pathology [18]. Likewise, vascular smooth muscle cell (VSMC) proliferation and calcification are among the critical cellular aspects that precipitate or exacerbate hypertension [19]. Other players, however, play a protective role in preventing the cardiovascular complications of hypertension and heart failure. These include the cardiac hormone atrial natriuretic peptide (ANP) [18]. It is the interplay between these various adverse and protective mediators that constitutes a framework of hypertension pathophysiology (Fig. 1).

It is well-established that hypertension also has a solid genetic basis [20]. Generally, hypertension evolves as a combined effect of several factors in the endocrine, renal, and cardiovascular systems. This is especially well-documented in obese people suffering from metabolic syndrome, a phenomenon of extensive genetic interplay [21]. This underpins the importance of genetic input into disease phenotype. More recently, an epigenetic link between molecules like MicroRNA (miRNA) and the evolution of hypertension, both primary and secondary, has been highlighted [22]. In this manuscript, we aim to review the experimentally determined associations between miRNA molecules and hypertension treatment. This is embodied by the physiological responses to antihypertensive drugs due to epigenetic mechanisms, i.e., the pharmaco-epigenetics of hypertension, with a focus on miRNA effects.

## Epigenetics and hypertension

### DNA methylation

DNA methylation is an example of an epigenetic mark that is responsive to environmental cues and is mitotically stable. It is associated with a range of biological processes, including those involved in the development of hypertension and stroke [23–25]. Candidate gene studies in animals and cell lines have demonstrated the role of DNA methylation in the pathogenesis of hypertension, including the HSD11B2 gene [26]. In humans, DNA methylation has been linked to hypertension for this gene as well [27].

### Histone modification

Histone modification is known to be one player in the regulation of vascular function in hypertension. That is not surprising given that histones play a crucial role in maintaining chromatin structure and regulating gene expression. One of the five histones found in eukaryotic nuclei, histone 3 (H3), has an N-terminal tail that can be modified by methylation or acetylation of lysine and arginine residues, as well as phosphorylation of serine and threonine residues [28]. Histone acetyltransferases (HAT) add acetyl groups to histones, while histone deacetylases (HDAC) remove them. The effects of these modifications can vary depending on the residue or moiety that is modified.

### Non-coding RNAs

Advances in the field of epigenetics have revealed some of the missing pieces in understanding the hereditary puzzle, which can explain why the same genome can lead to different phenotypes without changes to the primary DNA structure. The elusive factor in comprehending the complex and multifactorial nature of hypertension may be the non-coding portion of the human genome [29]. Previously, it was widely believed that all human genes coded for proteins, but it is now known that more than 95% of these genes do not produce proteins. Instead, they are transcribed into non-coding RNA (ncRNAs) molecules, which have vital roles in regulating protein-coding genes [30].

The purpose of this review is to provide an overview of the current understandings for the role of miRNA in the intricate regulatory processes involved in the pathophysiology of hypertension.

## MicroRNA and hypertension

**Fig. 2:**
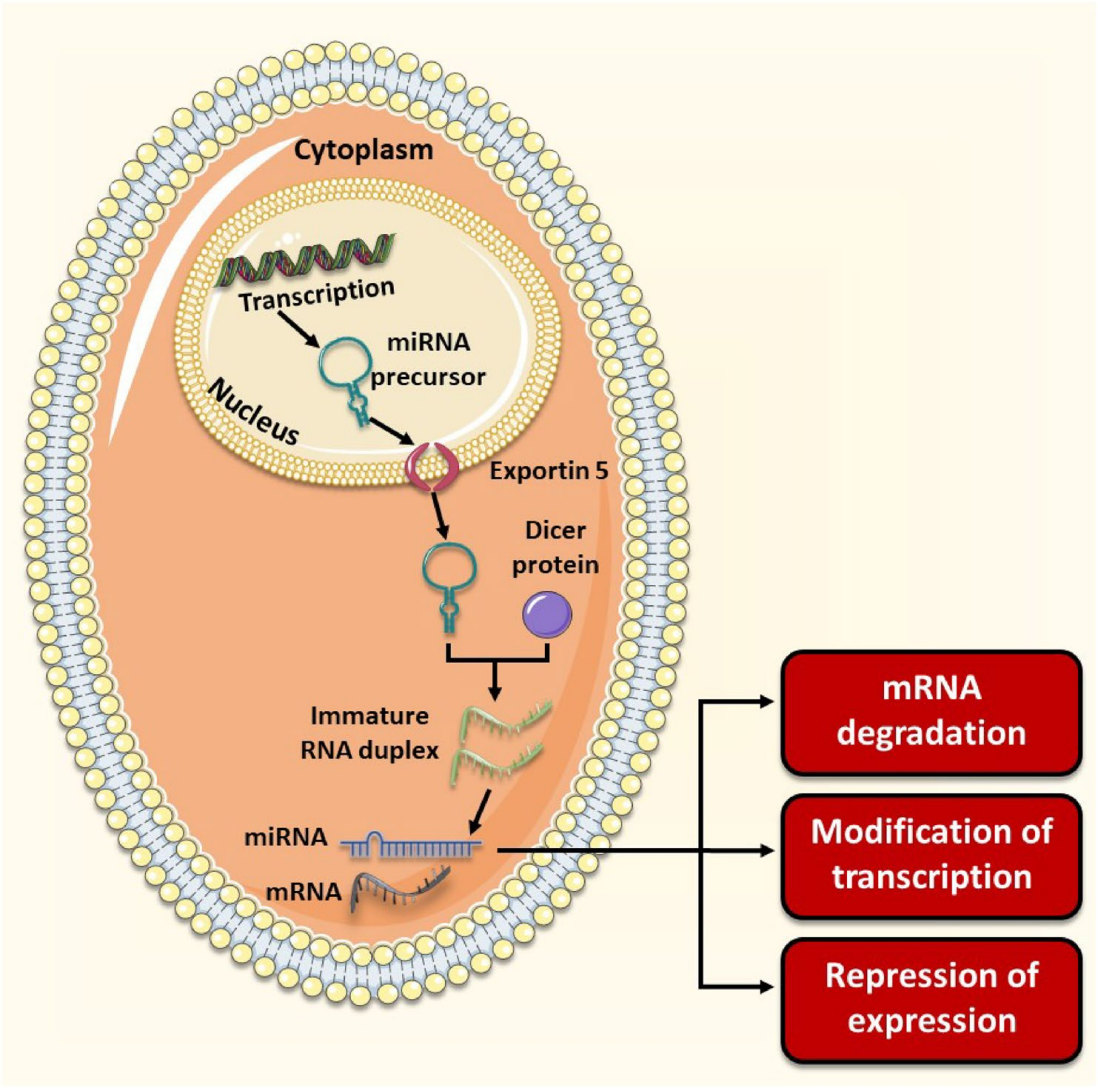
Transcription of miRNA begins in the nucleus. A miRNA precursor is initially yielded from transcription. It is then transported from the nucleus via the nuclear envelope using the exportin 5 molecule. The resultant miRNA precursor molecule in the cytoplasm is transformed into a double-stranded form via the DICER proteins in an ATP-dependent fashion. Finally, the mature miRNA molecule strand can exercise its degradative, repressive, or even in some cases translation activities

MicroRNAs are molecules transcribed from DNA into “non-coding” single-stranded RNA molecules [31] (Fig. 2). These molecules exhibit short, conserved sequences that function as modulators of gene expression [32]. This modulation is achieved by binding the 3’UTR of the mRNA target, thereby regulating its translation [33]. Gene expression regulation is exhibited by mRNA strand de-adenylation, degradation, and inhibition of the ribosomal apparatus assembly, among other mechanisms that remain to be fully elucidated [34].

A plethora of miRNA molecules have been linked with the emergence of traditional primary hypertension [35]. They have also been correlated with pulmonary hypertension and pre-eclampsia [36]. In fact, some miRNA molecules may predict the development of hypertension (e.g., miRNA 4516 upregulation and miRNA 145 downregulation) [36]. For instance, diminished levels of miRNA-21 were strongly correlated with ameliorated arterial stiffness in patients with well-controlled essential hypertension [22, 37]. Hence, miRNA-21 may serve as a potential prognostic predictive marker and target for therapy [22, 37]. Moreover, other molecular non-coding RNA agents still need further studies to be assertively dubbed a protective or harmful mediators with regards to a hypertensive phenotype. This is the case with molecules like miRNA-92a-3p, which is proposed to have a positive correlation with systolic and diastolic blood pressure measurements, despite having a negative association with occupational noise exposure, a contributor to hypertensive events [38].

Genetic pre-disposition towards hypertension is becoming increasingly unraveled as more research on the matter prevails. Nearly 2 percent of our entire genome pertains to coding regions that deal with blood pressure regulation. The field of exploring the role of non-coding RNA molecules has thus been extensively revamped. However, several limitations, including verifiable modalities by which efficient molecular standardization in these studies, exist and are being investigated [39, 40]. Nevertheless, further studies delineating the roles of all non-coding RNA molecules may serve as an attractive avenue for potential prevention of the progression of hypertension [41].

### MicroRNAs and essential hypertension

The evolution of hypertension is underpinned by inflammatory processes [42]. This includes vascular inflammation, endothelial dysfunction, reactive oxygen species (ROS) production secondary to a plethora of dynamic factors [43–50]. Moreover, as will be discussed in this paper, miRNA is linked to upholding and maintaining these inflammatory processes [51]. An example of this is miRNA-122 which perpetuates cardiovascular fibrosis mechanisms by downregulating several RAAS molecules such as ACE2, or agents from other systems that mediate hypertension when altered, such as apelin [52]. Similarly, other miRNAs such as miR-155, miR-212, miR-21, miR-19a/b and miR-20b mediate inflammatory processes that portend hypertension [53]. These miRNA molecules are active agents in the development of hypertension because of their involvement in various fibrotic vascular processes.

The Renin-Angiotensin-Aldosterone System (RAAS) is an important neurohormonal modulator of blood pressure and volume status [54]. Angiotensin II and aldosterone are pertinent mediators [55]. Angiotensin II helps in facilitating arterial vasoconstriction, especially in the context of blood loss. The autoregulatory RAAS helps maintain sodium balance over various fluid intake states with minor blood pressure alterations [54]. A less studied pathway is that of apelin and its associated proteins [52]. Indeed, it is intimately interlinked with the RAAS pathway so much so that ACE2 modulates its levels [56]. This apelin acts as an endothelial vasodilator and functions through the eNOS pathway, with a documented interplay with the MAPK pathway [57]. The following section portrays how miRNA molecules are physiologically related to RAAS and hypertension evolution or amelioration.

#### MiRNA-181a

**Fig. 3:**
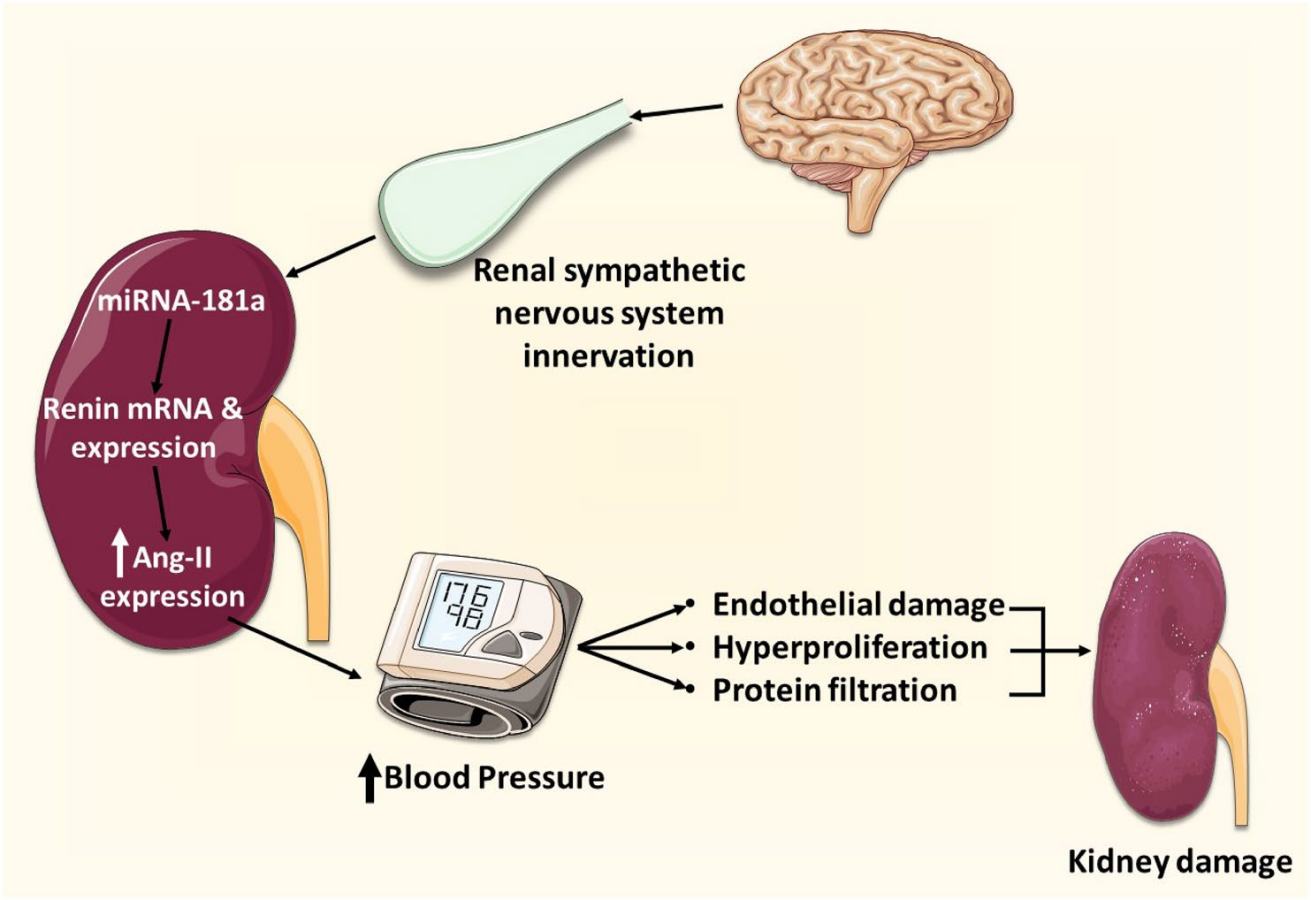
SNS innervation of the kidney induces decrease in miR-181a levels which activate the RAAS and raise blood pressure levels virtue of RAAS molecules such as Angiotensin II. This in turn can deteriorate kidney function, further exacerbating a hypertensive state

An excessively active sympathetic nervous system generally predisposes to a hypertensive state [34]. This is due to the upregulation of the RAAS system [58] (Fig. 3). Renal hyperactivation by the sympathetic nervous system is known to predispose to excessive renin secretion. This process is potentially mediated by miRNA [58]. For instance, relatively lower miRNA-181a, which is a negative regulator of Ren1 mRNA, leads to a hypertensive state in BPH/2 J mice [58]. Bilateral Renal denervation was shown to amplify miRNA-181a levels and its transcription factor Tcf7l2 such that hypertension in BPH/2 J mice was reversed [34]. Hence, renal sympathetic nerves contribute to the downregulation of miR-181a, a derivative to RAAS overactivity [59]. These findings support the inverse relationship between miRNA-181a and hypertension development in the context of an activated sympathetic nervous system.

#### MiRNA-132 and miRNA-212

In cases of *in-vivo* angiotensin II-induced hypertension, miRNA molecules such as miRNAs 132 and 212 were found to be elevated in the heart, kidney, aortic wall tissues of rats [60]. The activation of the G_αq_-coupled endothelin receptor was found to elevate the levels of these two miRNA molecules. Contextually, a decrease in these two molecules was noted after administration of Angiotensin II type 1 receptor (AT1-R) blockers, as opposed to treatment with beta-blockers [60]. Subsequently, one can deduce a correlation between these two miRNA molecules and a hypertensive status in response to angiotensin receptor blockers. Moreover, miRNA-132 has additional implications in the realm of cardiovascular morbidity [60]. MiRNA-132 might also be involved in the metabolic and CVD implications of frank obesity [61]. Studies with large sample sizes are needed to correlate miRNA-132 levels in subcutaneous tissue and hypertension in these individuals [61]. These findings all underscore a need for further research to elucidate underpinned relations between these miRNAs and hypertensive sequelae.

#### MiRNA-483-3p

Angiotensin II-induced activation of AT1-R in VSMCs elicits a miRNA expression signature. For instance, AT-II regulates miRNA 483-3p amongst other molecules [62]. Multiple proponents of the RAAS are targeted by miRNA-483-p, including angiotensinogen and angiotensin-converting enzyme 1 (ACE-1), in VSMCs [62]. Binding sites of this miRNA include RAAS genes AGTR2, ACE-1 and 2, and AGT. Thereby, RAAS homeostasis coordination is achieved. Moreover, miR-483 expression is decreased in the sera of patients with idiopathic pulmonary hypertension, peculiarly in more severe cases [63]. Overexpression of miRNA-483 in pulmonary arterial endothelial cells inhibits the genes related to pulmonary arterial hypertension [63]. Additional findings indicate that miR-483-3p has a pertinent protective impact on endothelial cell functionality during hypertension onset [64]. MiRNA-483 may be a future therapeutic target with regards to CVD sequelae within the context of hypertension [64].

#### MiRNA-155

In the context of vascular remodeling diseases, miRNA-155 has been shown to be a promising target in ameliorating RAAS-mediated VSMC proliferation [65]. This is due to AT1-R being identified as an important miRNA-155 target in the context of mouse VSMCs [65]. This finding reveals the role of miRNA-155 in mediating anti-proliferative effects.

### MicroRNAs and pre-eclampsia

**Fig. 4:**
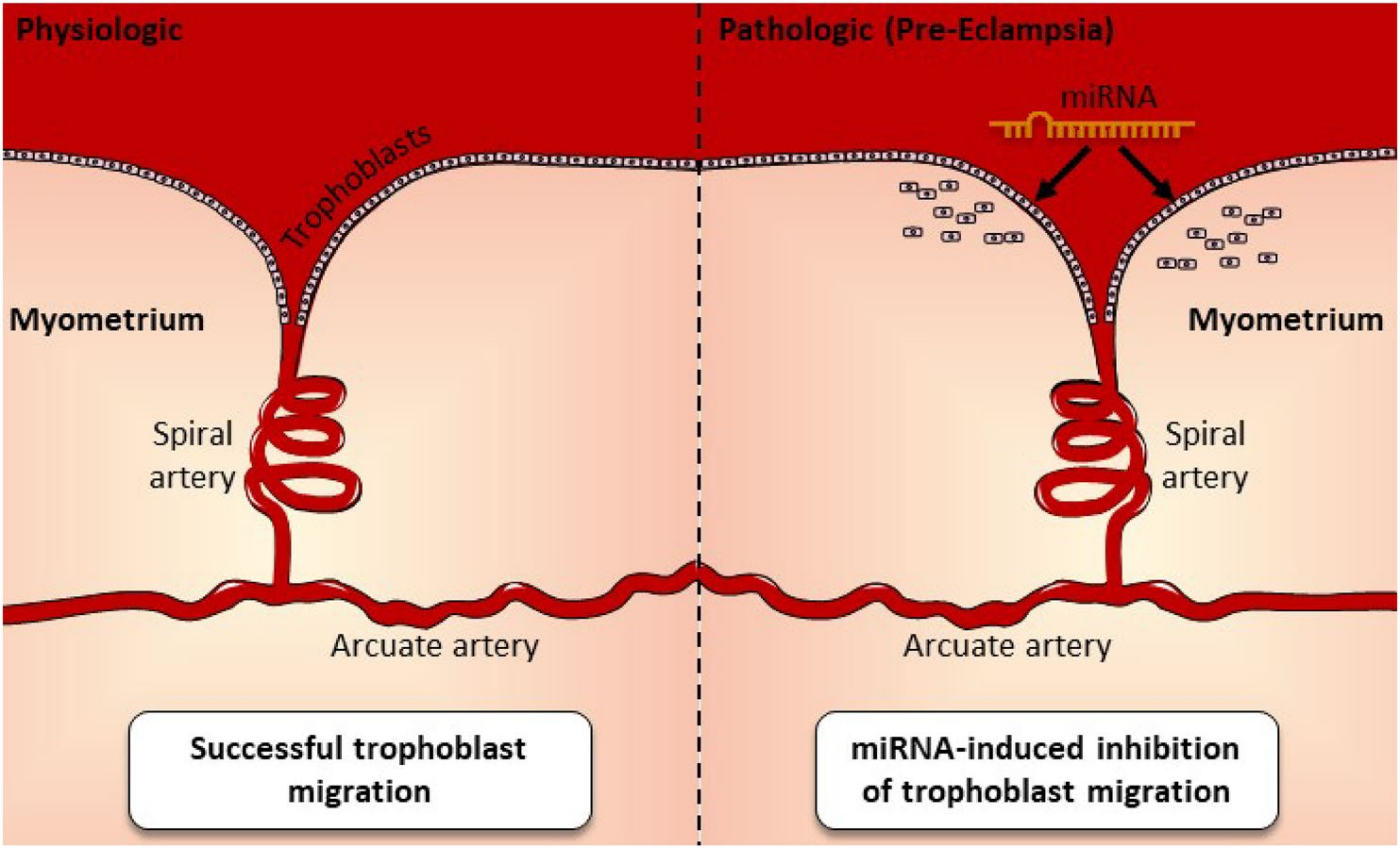
Depiction of one aspect of pre-eclampsia: trophoblast migration dysfunction. This dysfunction is elicited by microRNAs such as miRNA-210. Shallow trophoblast invasion is seen, resulting in unconverted narrow spiral arteries. This leads to fetal hypoxia virtue of endothelial injury. Sequelae of this hypoxia includes maternal hypertension, edema, and proteinuria

Pre-eclampsia is a disorder of pregnancy that consists of hypertension and proteinuria which occur after the 20th week of the gestational period [66]. It causes significant burden in terms of maternal and neonatal morbidity and mortality [67, 68]. Dysregulation of miRNA molecules have been posited to play a role in the development of pre-eclampsia (Fig. 4) [69]. Some of the miRNA molecules involved in the pathogenesis of pre-eclampsia shall be discussed below.

#### MiRNA-210

MiRNA-210 is one of the most studied miRNA molecules. Its role in the pathophysiology of pre-eclampsia has been explored [70]. MiRNA-210 was seen to be elevated in women with pre-eclampsia [71]. It has been isolated in the placenta and blood of patients with pre-eclampsia [71]. Its effect on trophoblast function (Fig. 4), mitochondrial function, and iron metabolism has been explored [69]. Generally, miRNA-210 expression is increased in hypoxic states. In the context of pre-eclampsia, miRNA-210 plays a role in the incessant inflammation seen. [69]. This is achieved by compromising mitochondrial function, thus stabilizing hypoxia-inducible factor -alpha (HIF-alpha) due to development of reactive oxygen species (ROSs). The expression of miRNA-210 is upregulated due to TLR-3 activation, which also stabilizes HIF-alpha, amongst a cascade of other molecular effects [69]. These findings point to miRNA-210 being deeply involved in pre-eclampsia development.

#### MiRNA-155

Endothelial nitric oxide synthase (eNOS), a major element in endothelial cell permeability, is regulated by miRNA-155 [72]. This has an implication in endothelial cell function and integrity in the context of pre-eclampsia [72]. In addition, vasodilation homeostasis in human umbilical vein endothelium was seen to be regulated by miRNA-155 [69]. The inhibition of this miRNA-155 molecule was observed to aid in the improvement of endothelial dysfunctional activity [69]. Hence, the role of miRNA-155 potentially involves the development of pre-eclampsia. Further studies are required to mark the pattern of miRNA-155 regulation in the context of pre-eclampsia.

#### MiRNA-124

The increased expression of miRNA-124-3p inhibits the pro-apoptotic effect of Ang-II, in addition to its role in ROS production in Human umbilical vein endothelial cells (HUVECs) [73]. This is accomplished by targeting EGR1 [73]. This finding reveals a pertinent role of miRNA-144-3p in mediating hypertensive outcomes.

### MicroRNAs and pulmonary arterial hypertension

**Fig. 5:**
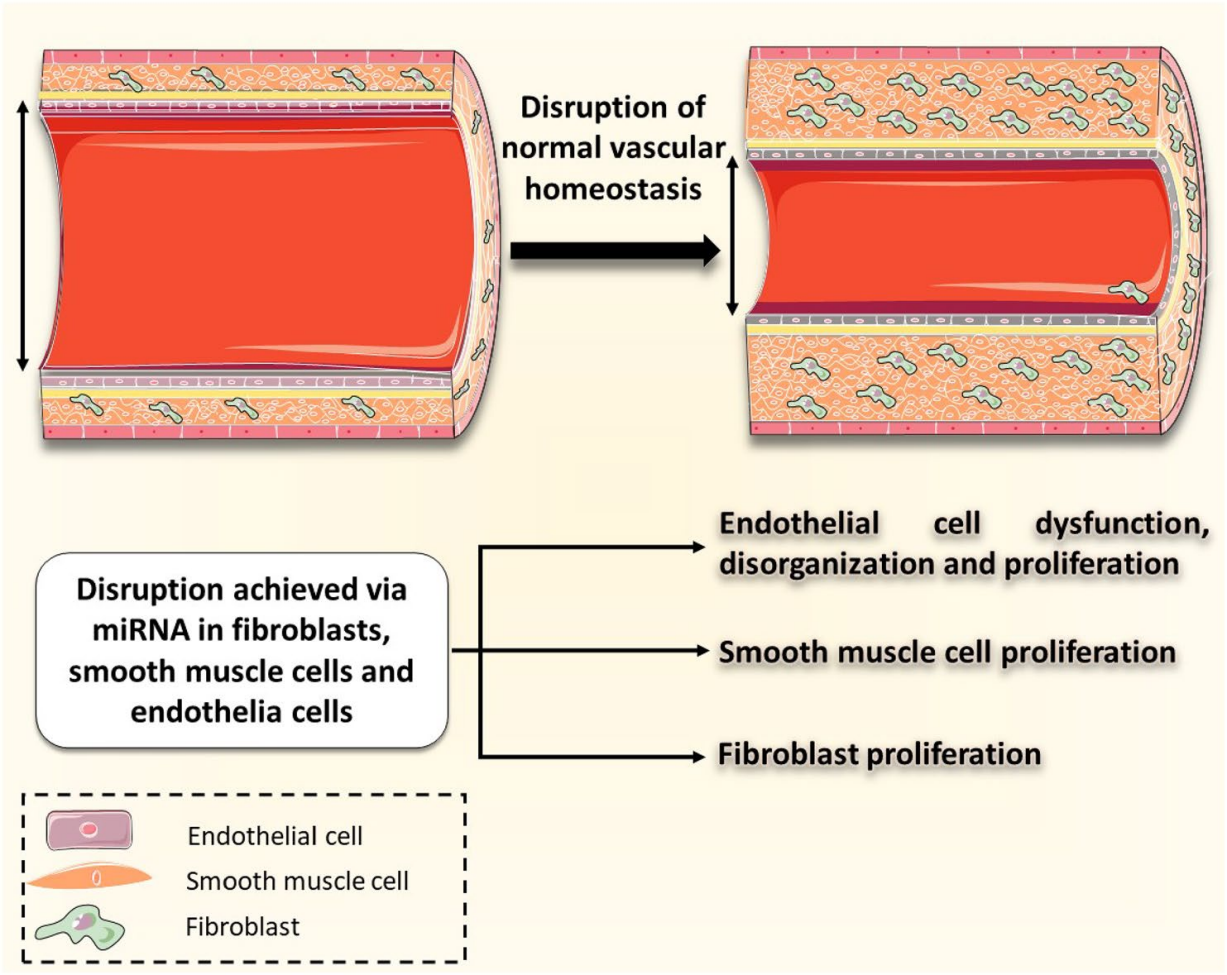
Vascular homeostasis is dictated by the phenotype and integrity of various vascular wall elements. This includes SMC, EC, and myofibroblasts. Dysfunction and hyper-proliferation of these elements disrupts the mentioned homeostasis, leading to the manifestation of Pulmonary hypertension. Various miRNA molecules have been implicated in this disruption. These include miRNA in endothelial cells (miR-17-5p, 20a, 27, 424, 503), miRNA in SMC (miR-17-5-p, 20a, 27, 124, 138, 145, 190, 204, 206, 210), and miRNA in fibroblasts (miR-124, 150)

Pulmonary arterial hypertension is a variant of hypertension that has significant burdens. It has unfavorable prognostic morbidity and mortality indicators [74]. Its pathogenesis arises via several mechanisms [75]. On a molecular basis, this entails the proliferation of pulmonary endothelial cells and smooth muscle cells, in addition to the latter’s migration and activation [76]. Many miRNA molecules have been implicated in the pathophysiology of this disease [77]. In fact, pulmonary arterial hypertension shares similar inappropriately activated pathways with cancers [78]. These activated pathways lead to the excessive proliferation and survival pulmonary arterial smooth muscle cells (PASMCs) within the pulmonary arterial wall and resultant lumen restriction [78]. The roles of miRNA-204 and miRNA-206 in PASMCs proliferation has been implicated [78]. Others such as miRNA-145, miRNA-21 and the miR17/92 cluster have been linked with the altered BMPR2 pathway [78]. Some of these miRNA molecules, and others, shall be discussed below (Fig. 5).

#### MiRNA-21

MiRNA-21 is thought to play a potential role in hypoxia-induced PASMC proliferation [22]. Indeed, upregulation of this molecule has been found to be facilitated by BMPR2 expression, whereby the miRNA-21 molecules also inhibit BMPR2 expression in a negative feedback loop. As such, it serves as a protective marker especially that its loss leads to Rho-Kinase activity upregulation, furthering pulmonary hypertension progression. [76]. This is another example whereby a miRNA molecule may alter the balance of hypertension development, here in the context of pulmonary hypertension. However, more research is needed to better elucidate the role of miRNA-21 in the context of resistant hypertension [79].

#### MiRNA-124

MiRNA-124 is downregulated in hypoxic conditions, and when overexpressed it can inhibit PASMC proliferation [76]. This may be capitalized on in future potential treatment modalities, particularly because miRNA-124 inhibits the NFAT activity as well as the subsequent transcription of interleukin-2, a key culprit in hypertension [76]. Furthermore, reduced levels of miRNA-124 has been reported in fibroblasts of patients suffering from pulmonary arterial hypertension [76]. This has an implication for its role in the migration and hyperproliferation of the respective fibroblasts [80], and thus in the pathogenesis of pulmonary hypertension. It appears that one of the underlying mechanisms of action employed by miRNA 124 is suppressing MCP-1 and PTBP-1, which in turn regulate a series of signaling pathways, some of which involve fibroblast proliferation [76]. Thus, this miRNA molecule may serve as a protective factor in the context of pulmonary hypertension [81].

#### MiRNA-210

As discussed previously, miRNA-210 is upregulated in the context of hypoxia in several cells including PASMCs [76], and apparently this increase is facilitated by the HIF-1 pathway. This upregulation leads to the suppression of E2F3, thus increasing the resistance to apoptosis. Eventually, this promotes PASMC hyperplasia, [76], clearly making it a culprit in exacerbating pulmonary hypertension.

#### MiRNA-150

MiRNA-150 levels have been seen to be diminished in patients with pulmonary hypertension. Moreover, they have been correlated with a rather poor survival [82]. This was also observed in the plasma and pulmonary cells of rats treated with hypoxia [82]. MiRNA-150 upregulation relieves this and suppresses hypoxia-driven collagen fibrous formation, and the expression of markers such as α-SMA, TGF-β1, and collagen I in pulmonary arterial smooth muscle cells and lung tissues [82]. In addition, miRNA-150 upregulation represses the excessive proliferation of these PASMCs driven by hypoxia via the AKT/mTOR signaling pathway. Moreover, this upregulation inhibits proliferation and resistance to apoptosis in relevant endothelial cells [82]. These findings indicate that miRNA-150 is a promising potential pulmonary hypertension ameliorator. Moreover, this molecule acts as an independent prognostic survival indicator [83]. In addition to its previously mentioned proliferation attenuating effects, miRNA-150 alters phospholipid signaling, with PTPMT1 mostly affected [83]. PTPMT1 reduces inflammatory activity, apoptosis and improves mitochondrial function in pulmonary endothelial cells and progenitors in the context of pulmonary hypertension [83]. These effects are mediated by diminished expression of pro-fibrotic, pro-apoptotic, and pro-inflammatory genes. These genes include *c-MYB*, *NOTCH3*, transforming growth factor β (*TGF-β*), and *Col1a1*. Mi-RNA1-150 thus serves as a major effector in pulmonary hypertension [83].

#### MiRNA-140-5p

In the context of pulmonary hypertension, reduced levels of miRNA-140-5p molecules were observed [84]. The therapeutic implications of replacing this miRNA were shown. MiRNA-140-5p targets the E3 ubiquitin ligase Smurf1. The regulation of the latter is a key in the context of pulmonary hypertension [84]. Smurf1 itself targets the BMPR2 molecule, the inactivation of which is the main key implicated in causing of pulmonary hypertension. The restoration BMPR2 signaling, by exogenous delivery of this miRNA molecule, and thus targeting Smurf1, is a promising therapeutic goal of this condition [85].

## MicroRNAs and responses to antihypertensive drugs

Various antihypertensives have been used over the past several decades to ameliorate hypertensive outcomes [86]. These include ACE-inhibitors, angiotensin receptor blockers (ARBs), aldosterone antagonists, in addition to alpha and beta-blockers, and calcium channel blockers [87]. Recently, miRNA molecules have thus been found to affect the pharmaco-epigenetic basis of antihypertensives [88]. This has vast implications on the response and treatment-resistance in individuals on antihypertensives [88]. This is pertinent given that half of the patients on anti-hypertensives do not have controlled hypertension [89]. The following section shall address the pharmaco-epigenetic relationship between miRNA molecules and the currently available antihypertensive treatment modalities (Table 1).

**Table 1:** Most widely utilized drugs in hypertensive diseases, the mechanisms and examples of which are outlined

Classes	Examples	Mechanism of action
ARBs	Valsartan; losartan	Block ATII receptors
ACE inhibitors	Enalapril; lisinopril	Inhibit ACE
Beta-blocker	Labetalol; metoprolol	Block beta receptors
Alpha blocker	Terazosin; doxazosin	Block alpha receptors
Calcium channel blockers	Nicardipine; amlodipine	Block calcium channels
Diuretics	Thiazides: *hydrochlorothiazide*, Loop diuretics: *Furosemide*	Promote renal-based diuresis

### Overview of candesartan in hypertension

Candesartan is an ARB and one of the most effective treatments of hypertension [37]. As the name implied, one main mechanism by which it mediates its action is via inhibiting the action of angiotensin II, a main effector of the RAAS. This ameliorates the burden of excessive RAAS activation [90]. It is particularly effective in individuals who have unfavorable side effect outcome (e.g., excessive dry cough) when exposed to ACE-inhibitor [91]. The absence of this widely noted side effect with ARBs is due to the lack of inhibition of substance P and bradykinin degradation [91]. Findings related to epigenetic involvement within candesartan response shall be discussed below.

#### Pharmaco-epigenetics of candesartan

Candesartan has been confirmed to ameliorate hypertensive sequelae via several mechanisms [92]. One of the mechanisms is by preventing angiotensin II–induced vascular smooth muscle cell proliferation [37]. This effect is mediated by alterations of several miRNAs [37]. Notably, miRNA-301b is shown to be a main target of candesartan [37]. It prevents the decrease of miRNA-301b and thus mediates an anti-proliferative action. Furthermore, studies show that inhibition of this miRNA specifically seems to minimize the physiologic effects of candesartan [37]. Thus, the inhibition of SMC proliferation is lifted [37]. MiRNA-301b prohibits SMC proliferation via targeting the 3’UTR of STAT3, preventing its expression and thus role in the cell cycle G1/S transition [37]. These mechanisms delineate the role of candesartan and its respective miRNA counterpart in ameliorating vascular wall alterations in hypertension.

### Pharmaco-epigenetics of beta-blockers

Beta-blockers alleviate hypertension by reducing renin release [93]. This is facilitated by antagonizing beta-1 receptors on juxtaglomerular renal cells [94]. In addition, beta-blockers reduce heart rate by antagonizing beta-1 receptors on cardiac cells [95]. Patients that exhibit RAAS-mediated increase in blood pressure benefit the most from beta-blocker treatment. This response to beta-blocker treatment has been shown to be manipulated by plasma miRNA [96]. This reveals a pertinent need to investigate the relationship between miRNA molecules and beta-blocker treatment.

MiRNA 19a was shown to be a possible biomarker of beta-blocker effectiveness in the setting of patient’s selective response to antihypertensive [94]. This is mediated by several points of regulation in the beta-adrenergic signaling pathway: ADRB1 (the beta-blocker receptor protein target), beta-adrenergic kinase, adenylyl cyclase, and others [94]. In fact, beta-blocker treatment may induce miRNA 19a expression which subsequently downregulates ADRB1 expression [94]. Moreover, molecules such as miRNA-101 and miRNA let-7e have been posited to modulate antihypertensive beta-blocker response [94]. More research is needed to elucidate the exact mechanistic response of these miRNAs to beta-blockers.

#### MicroRNA: dictating interpersonal beta-blocker effectiveness

Different beta-blocker classes elicit various responses [97]. Atenolol and Nebivolol have similar effects on blood pressure and heart rate. However, they differ in their effects on left ventricular integrity in rodent-models of hypertension. This is through differing effects on miRNA molecules [98]. Nebivolol was shown to better ameliorate left ventricular systolic function decline. In addition, it decreases the burden of ventricular fibrosis and remodeling [98]. This is achieved by preventing the decrease in levels of miRNA-27a and miRNA-29a (which target Sp1) in rodent receiving high-salt diet, in addition to miRNA-133a (which targets Cdc42) [98]. These findings portray the various effects of miRNA molecules in affecting therapeutic drug response in addition to differential drug effects even within the same class of beta-blockers.

### MicroRNA in the context of dyslipidemia and hypertension

Dyslipidemia involves an unfavorable lipid profile [99]. This entails high plasma low-density lipoprotein cholesterol (LDL-C), triglycerides (TGs), total cholesterol (TC), and reduced high density lipoprotein cholesterol (HDL-C) [100]. Atherosclerosis is a long-term sequela of dyslipidemia [101, 102]. This phenomenon eventually disrupts blood flow in affected blood vessels [103]. This disruption induces a pro-inflammatory state [104]. Peculiarly, it represses protective factors such as eNOS [103]. This process has been shown to be regulated by miRNA involvement [103]. The development of atheromatous plaques leads to potentially lethal consequences such as myocardial infarction (MI) [105]. This appears to be influenced by miRNA molecules, such as miRNA-10a, -126, -145a/b, -185, -210, and -326 [103]. These molecules have been seen to be elevated inside these arterial plaques in comparison to arteries with no plaque [106]. Meanwhile, certain miRNA molecules appear to be protective [103]. These findings support the hypothesis that epigenetic mechanisms involving miRNA regulate not only hypertensive mechanisms in bringing about unfavorable cardiovascular outcomes, but also dyslipidemia-related sequelae.

#### Statin overview in context of microRNA

Statins are 3-hydroxy-3methylglutaryl-coenzyme A (HMG-CoA) reductase inhibitors [107]. They are a reliable choice in ameliorating unfavorable lipid profiles [108]. They hinder the progression of the lipid synthesis pathway. This is facilitated by preventing the formation of the mevalonate (MVA) intermediate via inhibiting the HMG-CoA reductase rate-limiting enzymatic step [107]. This decreases de novo cholesterol synthesis and increases the feedback expression of low-density lipoprotein receptors (LDL-R) in tissues like the liver. Thus, statins decrease the amount of circulating cholesterol in the blood [109].

It has been demonstrated that statins’ pleiotropic and adverse effects are mediated by miRNA molecules [110]. The pleiotropic effects include anti-inflammatory, antioxidant, anti-thrombotic, and anti-proliferative consequences on the blood vessel wall. This is pertinent to hypertension as it reduces the vascular remodeling profile [103]. The involvement of miRNA processes, however, in these latter pleiotropic effects of statins remains to be further explored in future research.

#### Pharmaco-epigenetics of statins

The response to statin therapy seems to be influenced by selective miRNA markers [110]. These markers regulate drug transporter and nuclear CYP450 receptors [111]. This is achieved by modulating CYP3A enzyme functionality and expression. Culprits of this regulation include miRNA-27b which affects CYP3A4 gene expression [103]. In fact, inter-individual discrepancies in statin (e.g., atorvastatin) metabolism is due to varying expression levels of miRNA-27b and miRNA-206 [103]. Moreover, miRNA-142 was affirmed to be a major variable in determining whether expression of CYP3A4 and CYP3A5 occurred. This is due to miRNA-142’s transcriptional silencing. MiRNA-133a has also been shown to play a role in mediating statin effects such as lovastatin in the context of endothelial cell function [103]. These findings reveal the pertinence of miRNA molecules in mediating statin effects and dictating its metabolism and therapeutic response.

### Aspirin and hypertension

Aspirin, the non-selective cyclooxygenase (COX) inhibitor, has been widely used for its effects as an antiplatelet drug at low doses (< 300 mg) [112]. It prevents platelet aggregation via preferential irreversible blockade of COX-1 inside platelets [113]. This prompts the need for the generation of new platelets to produce thromboxane A2 [114]. However, its usage as a potential management option in hypertensive patients has been discussed in the literature in recent years [115]. The relevant studies have been limited though, with discrepancies in factors like concomitant drug utilization and dosage of aspirin [114]. Aspirin’s benefits as a possible adjunct therapeutic agent in the context of hypertension also differ in outcomes with respect to males and females [114]. Future research is needed to elucidate aspirin’s feasibility as an option in modulating arterial vascular tone, amongst other blood pressure effectors [10].

#### Pharmaco-epigenetics of aspirin

The physiologic response to aspirin is regulated by certain miRNA molecules [116]. miRNA serve as markers for aspirin resistance, a condition that plagues one fourth of CVD patients [88]. MiRNA-135a-5p and miRNA-204-5p are implicated in this resistance. There are inter-individual differences in relative expression dictating varying response to treatment. These 2 miRNA molecules have been claimed to affect the expression of genes such as THBS1, CORO1C, CDC42, MAPRE2 [88]. Moreover, when indomethacin, a mimicker of aspirin in terms of effects, was used to monitor platelet reactivity, miRNA-19b-1-5p expression was decreased [116]. This serves as an instance of insensitive drug response. This qualifies miRNA to be a potential biomarker of aspirin resistance [88].

In addition, aspirin appears to prevent the abnormal proliferation and calcification of VSMCs [117], a marker of atherosclerotic disease. Contextually, this anti-proliferative and anti-inflammatory effect of aspirin in VSMCs is mediated, at least partly, by miRNA-145 [118]. When atherosclerotic plaques from patients treated with aspirin were compared with those from untreated patients, it was found that there is a significantly higher level of miR-145 and lower CD40 levels in plaques from the treated patients [118]. Moreover, aspirin’s role as an antiplatelet therapeutic agent also seems to be linked to miRNA regulation. For instance, aspirin robustly decreases the expression of miRNA-126 and miRNA-223 [119, 120]. These findings reveal the interlinkage between miRNA and responsiveness to aspirin.

#### Aspirin in pre-eclampsia: focus on microRNA

Endothelial cell dysfunction is an infamous contributor to the pathogenesis of the inflammatory gestational disease, pre-eclampsia. This is due to reduced eNOS/NO vasodilatory activity when the endothelium is compromised [121]. Aspirin has been shown to have a preventive effect with regards to pre-eclampsia [122]. Indeed, evidence shows that aspirin could abrogate TNF-α induced endothelial dysfunction by suppressing miR-155 [121]. This is another example whereby a hypertensive state is influenced by aspirin via modulating miRNA effects.

### Overview of clopidogrel in hypertension

Clopidogrel is an antiplatelet drug that serves to inhibit the activation and aggregation of platelets [90]. This is facilitated via the irreversible binding of active metabolites to the platelet P2Y12 variant of ADP receptors [90]. As is the case with aspirin, clopidogrel may have some utility in subsets of hypertensive patients [123]. Indeed, clopidogrel elicits a preventive effect on angiotensin II-induced inflammation and fibrosis of the heart [90].

Acute rises of blood pressure have been known to platelet activation, a phenomenon that clopidogrel inhibits [90]. It is also now recognized that hypertension is associated with inflammatory processes in vessel wall dynamics [124]. This is in part mediated by incessant platelet activation, particularly in patients with microalbuminuria and vascular lesions [125]. In addition, another major platelet inflammatory mechanism known as the platelet-leukocyte conjugation is a culprit in this process [126]. The nature of platelet involvement in vessel wall dynamics reveals a potential future use of clopidogrel in hypertension. However, future research is needed to elucidate the major potential beneficiaries of clopidogrel in modulating inflammation in hypertension and preventing adverse critical outcomes like MI.

#### Pharmaco-epigenetics of clopidogrel

Clopidogrel, as an antiplatelet, is a non-active prodrug. It is primarily absorbed in the intestine via ABCB1 transporters. It is then activated to a metabolite by CYP450 enzymes CYP2A4, CYP3A5, CYP2B6, CYP2C19, CYP2C9, and CYP1A2 [90, 127]. Irreversible ADP-receptor binding by way of this active metabolite thus prevents the activation of platelets [128]. However, conventional dosing of clopidogrel has shown, in a considerable percentage of recipients, to elicit a subpar antiplatelet response [90]. Pharmacogenomic bases of interpersonal differences in responses to clopidogrel by way of single nucleotide polymorphisms (SNPs) in certain CYP enzymes were not shown to account for the whole picture [90, 127]. On the other hand, miRNA molecule polymorphisms were shown to play a central part in these interpersonal differences in responses [90]. P2RY12 and CYP2B6 are targets of miRNA-605 [90]. Polymorphisms that give rise to miRNA-605 A/G instead of the G allelic variant prevent the maturation of the miRNA molecules. This prevents the aversion of unfavorable coronary syndrome outcomes in patients on clopidogrel [90]. In fact, this polymorphism may serve as a future biomarker in the context of predicting future events in patients on clopidogrel for the long-term [90]. These findings demonstrate the pertinent effect of miRNA molecules in altering clopidogrel response.

### Other therapeutic strategies

With the advent of advanced molecular technologies, miRNA involvement as pathogenesis mediators in many diseases has become more apparent. However, as delineated in this paper, some also serve as protective factors in hypertension. Thus, therapeutic modalities may make use of miRNA manipulation to maintain a balance at the cellular level with regards to gene regulation. In this section, potential miRNA therapies shall be discussed.

Chemically manipulated oligonucleotide molecules termed ‘antagomirs’ have been observed to target miRNA molecules and silence them effectively. The mode of delivery of these molecules remains an important obstacle to overcome in the future [129]. Nevertheless, this can herald several therapeutic outcomes. For example, utilizing intravenously given antagomirs conjugated to cholesterol is a possible mechanism [129]. Antagomirs against miRNA-21 were shown to block cardiac, renal, and pulmonary fibrosis [129]. Moreover, it was shown that targeting both miRNA-425 and miRNA-155 with anti-miRNAs is effective in modulating ANP levels, hence ameliorating hypertension [130]. Examples of targeting miRNA in alleviating pulmonary hypertension have been delineated [131]. This includes inhibiting miRNA-17 via an antagomir, which leads to the upregulation of the BMPR-2 pathway, thus ameliorating hypertension [132]. Moreover, findings have shown that restoring certain miRNA molecules in hepatic stellate cells may potentially alleviate intrahepatic portal hypertension [133]. Other molecules such as miRNA sponges, and locked-nucleic-acid oligonucleotides have also been studied in the context of diseases not isolated to the cardiovascular domain [129]. As a better understanding is attained with regards to miRNA chemical safety, stability, and delivery as a therapeutic modality, better outcomes can be reached [131].

#### Other considerations

**Fig. 6:**
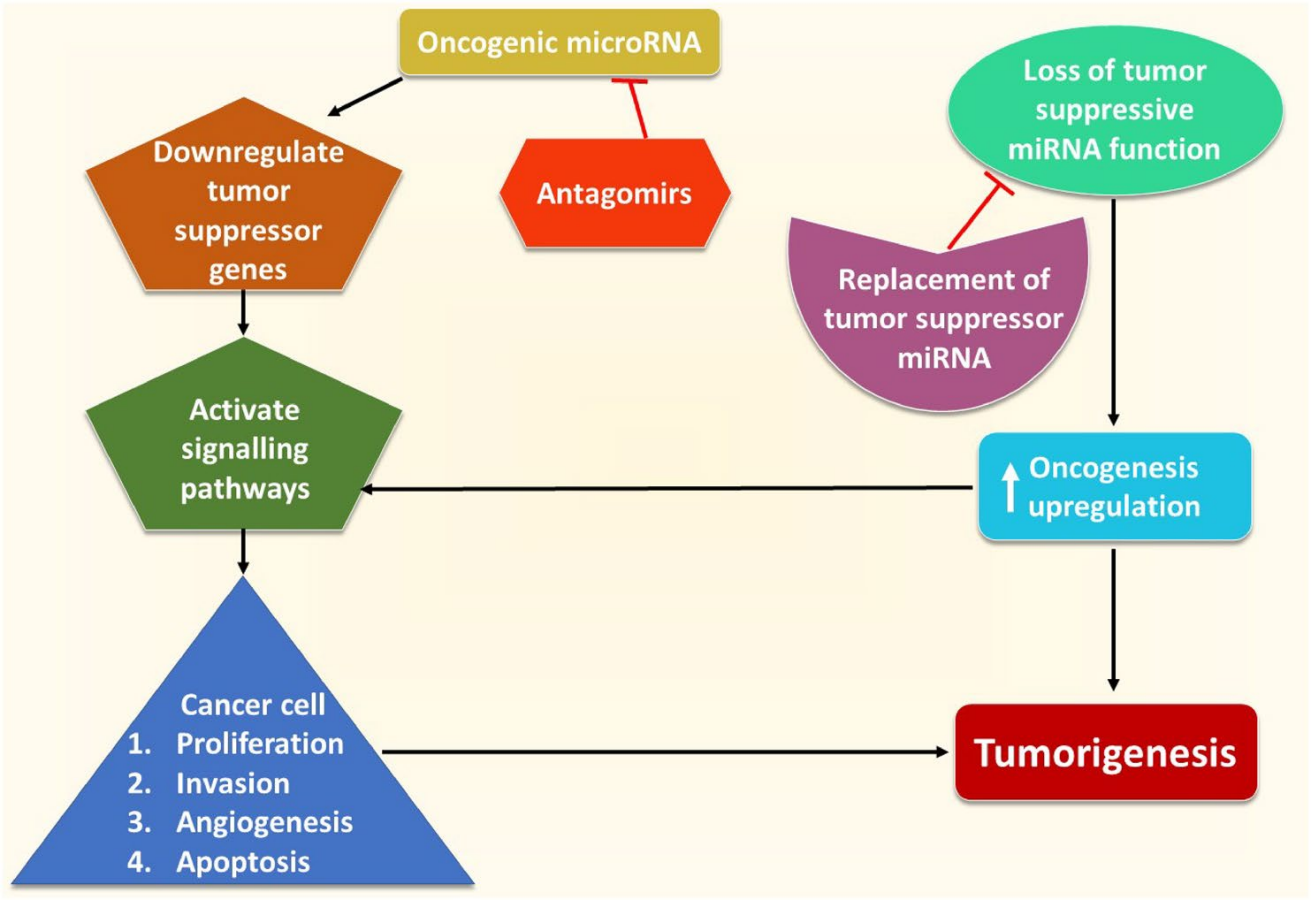
MiRNA molecules may aid in shifting the balance of cells towards an oncogenic phenotype. Others have a tumor suppressive function. Therapies focused on manipulating these individualized miRNA molecules’ respective function are on the rise. This includes antagomirs that can silence certain oncogenic miRNA. Moreover, synthetic miRNA can be utilized to replace miRNA with loss of tumor suppressor function. These can help prevent the propagation of signaling pathways that favor tumorigenesis

MiRNA molecules are involved in the pathogenesis of many diseases, including cancer. They are interlinked with cancer biological processes such as angiogenesis, apoptosis, proliferation, and invasion/metastasis [134]. In fact, they have been implicated with resistance that some cancer therapeutic regimens have [135, 136]. Cancer cells exhibit certain phenotypic profiles, through miRNA signatures; this can help overcome diagnostic and therapeutic dilemmas [137]. As a matter of fact, miRNA-based therapies have been studied in the context of breast cancer cases [138] (Fig. 6). These phenotypic markers are also exhibited in the context of spinal muscular atrophy through miRNA signatures as well [139]. This can also exhibit a form of biomarker tracking, which can help surmount therapeutic challenges. These encompass a few of the examples whereby a form of epigenetic signaling can make all the difference. Future genomic and epigenetic studies are required in each respective medical field to better help elucidate gaps in diagnostics and therapeutics.

## Conclusion

Pharmaco-epigenetics is a promising field in the context of personalized health optimization. It is one of the major effectors in predisposing to hypertension. Here, we delineated various means by which epigenetics plays its part, with a primary focus on how miRNA molecules could exacerbate, or ameliorate, hypertension. Moreover, antihypertensive drug response and resistance was seen to be drastically affected by miRNA. Further research is required to better elucidate the miRNA involvement in dictating antihypertensive outcomes. Moreover, additional studies in the field of epigenetics are needed to develop personalized antihypertensive pharmacologic treatment. Given the well-established therapies that already being utilized for hypertension, recognizing the pharmaco-epigenetic implications of miRNA molecules affecting the response to medications is vital for providing maximal treatment efficacy. Finally, given the loose associations between certain discussed treatment modalities (aspirin, clopidogrel, and statins) and hypertension, miRNA molecules may serve as a bridging gap in ultimately realizing the epigenetic understudied links of cardiovascular disease pathophysiology.

## Data Availability

No datasets were generated or analysed during the current study.
